# What do Cochrane systematic reviews say about non-surgical interventions for urinary incontinence in women?

**DOI:** 10.1590/1516-3180.2017.039420122017

**Published:** 2018-03-05

**Authors:** Anderson Adriano Leal Freitas da Costa, Igor Martins Vasconcellos, Rafael Leite Pacheco, Zsuzsanna Ilona Katalin de Jármy Di Bella, Rachel Riera

**Affiliations:** I Undergraduate Medical Student, Escola Paulista de Medicina - Universidade Federal de São Paulo (EPM-Unifesp), São Paulo (SP), Brazil.; II Undergraduate Medical Student, Escola Paulista de Medicina - Universidade Federal de São Paulo (EPM-Unifesp), São Paulo (SP), Brazil.; III MD. Researcher, Escola Paulista de Medicina - Universidade Federal de São Paulo (EPM-Unifesp), São Paulo (SP), Brazil.; IV MD, MSc, PhD. Gynecologist and Adjunct Professor, Department of Gynecology, Escola Paulista de Medicina, Universidade Federal de São Paulo (EPM-Unifesp), São Paulo (SP), Brazil.; V MD, MSc, PhD. Rheumatologist and Adjunct Professor, Discipline of Evidence-Based Medicine, Escola Paulista de Medicina, Universidade Federal de São Paulo (EPM-Unifesp), and Assistant Coordinator, Cochrane Brazil, São Paulo (SP), Brazil.

**Keywords:** Review, Evidence-based medicine, Therapeutics, Evidence-based practice, Urinary incontinence

## Abstract

**BACKGROUND:**

Urinary incontinence is a highly prevalent condition that impacts self-esteem and overall quality of life. Many non-surgical treatment options are available, ranging from pharmacological approaches to pelvic exercises. We aimed to summarize the available evidence regarding these non-surgical interventions.

**DESIGN AND SETTING:**

Review of systematic reviews, conducted in the Discipline of Evidence-Based Medicine, Escola Paulista de Medicina, Universidade Federal de São Paulo (EPM-UNIFESP).

**METHODS:**

A sensitive search was conducted to identify all Cochrane systematic reviews that fulfilled the inclusion criteria. Titles and abstracts were screened by two authors.

**RESULTS:**

We included 20 Cochrane systematic reviews: 4 assessing methods of vesical training, 3 evaluating pharmacological interventions, 4 studying pelvic floor muscle training approaches and 9 aimed at other alternatives (such as urethral injections, weighted vaginal cone use, acupuncture, biostimulation and radiofrequency therapy). The reviews found that the evidence regarding the benefits of these diverse interventions ranged in quality from low to high.

**CONCLUSIONS:**

This review included 20 Cochrane systematic reviews that provided evidence (of diverse quality) for non-pharmacological interventions for patients with urinary incontinence. Moderate to high quality of evidence was found favoring the use of pelvic floor muscle training among women with urinary incontinence. To establish solid conclusions for all the other comparisons, further studies of good methodological quality are needed.

## INTRODUCTION

Urinary incontinence (UI) can affect up to 50% of women worldwide.^1^ This rate can reach 77% among elderly women living in nursing homes.^2^ Additionally, recent research showed that 37.5% of younger women (from 30 to 50 years) in a primary care setting complained of stress incontinence.^3^

Urinary incontinence can be classified into three main types: stress, urge and overflow incontinence. Many women have mixed characteristics of more than one type.^4^^,^^5^^,^^6^ Identifying the classification can be useful for making decisions regarding therapy, since different therapeutic options are available for each type.^7^

Women presenting stress incontinence (the most common type in younger women) have involuntary leakage of urine relating to increased intra-abdominal pressure (e.g. during physical exercises, sneezing, coughing or laughing) in the absence of a bladder contraction.^4^ Urge incontinence (or “overactive bladder with incontinence”) is characterized by the urge to void immediately, preceding or accompanied by involuntary loss of urine, which can range from a few drops to full wetting of underwear.^4^ It is more common among elderly women and it is related to other clinical situations such as infections or chronic conditions (e.g. neurological disorders or diabetes). Overflow incontinence is characterized by continuous urinary leakage or dripping in the presence of incomplete bladder emptying. Women with overflow incontinence complain of weak or intermittent urinary stream, frequency, hesitancy and nocturia. When the bladder is full, stress leakage can occur or low-amplitude bladder contractions can be induced, thus leading to symptoms similar to urge or stress incontinence. It is caused by bladder obstruction or detrusor hypoactivity.^7^ Finally, women with mixed incontinence can present features of both stress and urge incontinence.^4^

It is important to point out that despite the considerable prevalence of UI, it continues to be an underdiagnosed and an undertreated disease: only about a quarter of women with complaints pursue medical care and, of those, less than 50% receive some type of treatment.^8^ Persistence of UI has been correlated with poor health outcomes comprising higher risk of urinary infections, sleep disturbances, falls and fractures, and depression.^9^^,^^10^

Because UI is a highly prevalent condition, with high impact on quality of life, several treatment options for it are currently available. These options range from pelvic exercises to surgical techniques, including pharmacological therapies and use of local devices.

The aim of the present study was to identify and summarize the evidence from Cochrane systematic reviews (SRs) regarding non-surgical therapeutic options for preventing urinary incontinence and for treating women with this condition.

## METHODS

### Design and setting

A review of Cochrane SRs was conducted within the Discipline of Evidence-based Medicine of Escola Paulista de Medicina, Universidade Federal de São Paulo (EPM-UNIFESP). This article was specifically developed for the section Cochrane Highlights, which is an initiative for disseminating Cochrane reviews. This initiative results from a formal partnership between the São Paulo Medical Journal and Cochrane, and it is supported by Cochrane Brazil.

### Inclusion criteria

#### Types of study

We included full Cochrane SRs published in the Cochrane Database of Systematic Reviews (CDSR). Protocols for SRs and withdrawn or outdated versions of SRs were not included. There was no limit on publication dates.

#### Types of participants

The participants considered were women who had been diagnosed with any type of urinary incontinence. We did not take into consideration reviews that included participants of both sexes but did not have any subgroup analyses that considered women alone.

#### Types of intervention

We considered SRs assessing any non-surgical therapeutic option, whether used in isolation or in combination with each other.

#### Types of outcomes 

We considered any clinical or laboratory outcomes, as evaluated by the authors of the SRs included.

### Search for reviews 

We conducted a systematic search in the Cochrane Database of Systematic Reviews (CDSR) (via Wiley) on October 18, 2017, sensitively using the MeSH term “urinary incontinence” and all related variants, in titles, abstracts and keywords. The detailed search strategy is presented in Table 1.

**Table 1:** Search strategy

#1 MeSH descriptor: [Urinary Incontinence] explode all trees
#2 MeSH descriptor: [Urinary Incontinence, Stress] explode all trees
#3 MeSH descriptor: [Urinary Incontinence, Urge] explode all trees
#4 MeSH descriptor: [Urinary Bladder, Overactive] explode all trees
#5 MeSH descriptor: [Urinary Bladder, Neurogenic] explode all trees
#6 (Urinary Incontinence) or (Incontinence, Urinary) or (Urinary Incontinence, Stress) or (Urinary Stress Incontinence) or (Incontinence, Urinary Stress) or (Stress Incontinence, Urinary) or (Urinary Incontinence, Urge) or (Urinary Reflex Incontinence) or (Incontinence, Urinary Reflex) or (Urinary Urge Incontinence) or (Urge Incontinence) or (Incontinence, Urge) or (Nocturnal Enuresis) or (Nighttime Urinary Incontinence) or (Incontinence, Nighttime Urinary) or (Urinary Incontinence, Nighttime) or (Diurnal Enuresis) or (Daytime Urinary Incontinence) or (Incontinence, Daytime Urinary) or (Urinary Incontinence, Daytime) or (Neurogenic Urinary Bladder) or (Bladder, Neurogenic) or (Neurogenic Bladder) or (Urinary Bladder Neurogenic Dysfunction) or (Neurogenic Dysfunction of the Urinary Bladder) or (Neurogenic Urinary Bladder Disorder) or (Neuropathic Bladder) or (Urinary Bladder Disorder, Neurogenic) or (Bladder Disorder, Neurogenic) or (Neurogenic Bladder Disorders) or (Neurogenic Bladder Disorder) or (Urinary Bladder Neurogenesis) or (Neurogenesis, Urinary Bladder) or (Bladder Neurogenesis) or (Neurogenesis, Bladder) or (Neurogenic Urinary Bladder, Atonic) or (Neurogenic Bladder, Atonic) or (Atonic Neurogenic Bladder) or (Neurogenic Urinary Bladder, Spastic) or (Neurogenic Bladder, Spastic) or (Spastic Neurogenic Bladder) or (Neurogenic Urinary Bladder, Uninhibited) or (Neurogenic Bladder, Uninhibited) or (Uninhibited Neurogenic Bladder) or (Overactive Bladder) or (Overactive Urinary Bladder) or (Bladder, Overactive) or (Overactive Detrusor) or (Detrusor, Overactive) or (Overactive Detrusor Function) or (Detrusor Function, Overactive):ti,ab,kw (Word variations have been searched)
#3 #1 or #2 or #3 or #4 or #5 or #6
Filter: in Cochrane Reviews

Filter: in Cochrane Reviews

### Selection of reviews

The titles and abstracts were screened by two authors (RLP, AALFC or IMV) independently. The SRs that met the inclusion criteria were selected. Any disagreement was resolved by consulting a senior author (RR).

### Presentation of results

The results from the search and the SRs included were presented through a descriptive approach (qualitative synthesis).

## RESULTS

### Search results

Our search strategy retrieved 106 references and, after screening the titles and abstracts, 31 SRs were preselected. After assessing full texts, 20 reviews were found to fulfill our inclusion criteria and were included for qualitative synthesis.

### Reviews included

In the following section, we present a brief individual summary of each review included, according to the intervention assessed. For details about the characteristics of the interventions, comparisons, outcomes and quality of evidence,^31^ see Table 2.

**Table 2:** Characteristics of interventions, comparisons, outcomes and quality of evidence

Pharmacological treatments				
Intervention	Comparators	Urinary incontinence type	Main findings	GRADE^**31**^
Adrenergic agonists^11^	Placebo	Stress	Adrenergic agonists seem to be better for:	Not assessed
			•reducing the number of pad changes	
			•reducing incontinence episodes	
			• improving symptoms	
Estrogen (systemic or local)^12^	Placebo	Not specified	Favors placebo:	Low
			•Improvement of urinary incontinence (oral systemic estrogen)	Low
			No difference between groups:	Moderate
			•Urinary incontinence episodes over 24 hours	Low
			•Women with urge incontinence (systemic estrogens)
			Favors estrogen:	Moderate
			•Women with urge incontinence (local estrogen)	Very low
			•Improvement of urinary incontinence (local estrogen)	
			•Improvement of urinary incontinence (local systemic estrogen)	
SNRI ^13^	Placebo and PFMT	Stress	Favors SNRI:	Not assessed
			•Higher proportion of participants with symptom improvement	
			Favors control group:	
			•Lower rate of adverse events	
			No difference between groups:	
			•Rate of cure	
Adding biofeedback and feedback to other therapies^14^	Other therapies without biofeedback	Urge and mixed	Favors addition of biofeedback:	Not assessed
			•Women’s perception of change in incontinence	
			•Women’s satisfaction	
Bladder training^15^	Pharmacological therapy, PFMT, biofeedback	Not specified	Favors bladder training:	Not assessed
			•Better patient perception of cure	
			•Improvement of quality of life	
Habit retraining^16^	No intervention	Not specified	No evidence available	Not assessed
Lifestyle interventions^17^	Any other intervention	Not specified	Favors weight loss program:	Low
			•Patient’s subjective impression of improvement	
			No difference between groups:	Low
			•Cure rates	Very low
			•Frequency of incontinence episodes per week	
Prompted voiding^18^	No intervention or other behavioral intervention	Not specified	Favors prompted voiding:	Not assessed
			Lower proportion of hourly checks that were wet	
			Fewer incontinent episodes	
Timed voiding^19^	Pharmacological and behavioral therapies	Not specified	No evidence available	Not assessed
Absorbent products^20^	Other absorbent product	Not specified	No evidence available	Not assessed
Acupuncture^21^	Pharmacological intervention (midodrine)	Stress	Favors acupuncture:	Not assessed
			•Improvement of symptoms (subjective assessment)	
			No difference between groups:	
			•Cure rate	
Mechanical devices^22^	No intervention, sham or other interventions	Not specified	No evidence available	Not assessed
Transurethral radiofrequency collagen denaturation^23^	Sham	Not specified	No difference between groups:	Low
			•Quality of life	
			•Adverse events	
			•Urinary tract infection	
Any intervention^24^	Different intervention, no intervention or placebo	Urinary incontinence after stroke	Favors meclofenoxate (compared with placebo):	Not assessed
			•Reduction of urinary symptom perception	
			Favors acupuncture (compared with usual care/no intervention):	
			•Reduction in number of participants with incontinence	
Urethral injection therapy^25^	Conservative or surgical management	Not specified	Favors injection:	Not assessed
			•Improvement of incontinence	
			•Quality of life (compared with conservative treatment)	
			Favors surgical treatment:	
			•Symptom improvement	
Weighted vaginal cones^26^	No treatment, PFMT, electrostimulation	Stress	Favors vaginal cones:	Not assessed
			•Incontinence cure (compared with no treatment)	
			No difference between groups:	
			•Subjective cure assessment (compared with pelvic exercises or electrostimulation)	
PFMT plus active treatment^27^	Active treatment alone	Stress, urge and mixed	•PFMT plus electrical stimulation versus electrical stimulation alone: no difference between groups regarding cure or improvement of symptoms	Low
			•PFMT plus heat/steam-generating sheet versus heat/steam sheet alone: benefit of PFMT regarding cure and symptom improvement	Moderate
			•PFMT plus vaginal cones versus vaginal cones alone: no difference between groups	Very low
PFMT	No treatment, placebo or sham^28^	Stress, urge and mixed	Favors PFMT: •Cure at end of follow-up	High
			•Symptom improvement	Moderate
	Different approaches for PFMT^29^	Stress, urge and mixed	•Perception of improvement: better with supervision twice a week than once a week No difference between groups:	Not assessed
			•Perception of improvement: no difference between group supervision and individual supervision, or between direct and indirect methods of PFMT	
PFMT in antenatal and postnatal period^30^	No treatment, different approaches for PFMT in antenatal and postnatal period^30^	Not specified	Favors intensive antenatal PFMT:	Not assessed
			•Incidence of incontinence over first six months after delivery (prevention)	
			Favors intensive postnatal PFMT:	
			•Rate of incontinence 12 months after delivery (treatment)	

PFMT = pelvic floor muscle training; SNRI = serotonin-noradrenaline reuptake inhibitor. *GRADE (Grading of Recommendations Assessment, Development and Evaluation) is a tool for assessing the quality of the body of evidence. Outcomes are classified as presenting high quality of evidence (high confidence that the estimated effect is close to the true effect); moderate quality of evidence (very likely that the estimated effect is close to the real effect, but there is a possibility that it is not); low quality of evidence (confidence in the estimated effect is limited); or very low quality of evidence (the true effect is likely to be substantially different from the estimated effect).

### Pharmacological interventions

#### Adrenergic agonists

This review^11^ aimed to assess the effects of adrenergic agonists for stress urinary incontinence (SUI), since these medications may improve bladder neck muscle contraction. The SR included 22 randomized clinical trials (RCTs) (1,099 participants) that assessed phenylpropanolamine, midodrine, norepinephrine, clenbuterol, terbutaline, phenylpropanolamine hydrochloride/diphenylpyraline hydrochloride and dabuzalgron (Ro 115-1240). The evidence was very limited and no data were pooled for primary outcomes. The results from single RCTs showed some possible effects from use of adrenergic agonists for improving symptoms and reducing the number of pad changes. However, these results were poorly reported and some important data were missing. Additionally, there is not enough evidence for assessing the effects of these drugs when compared with or combined with other treatments, and hence further larger trials with better quality are needed, to provide stronger evidence. The latest version of this review was published in 2010 and it was declared to be stable considering that some drugs were no longer used and that no new trials were expected.

For further details, refer to the original abstract, available from: http://onlinelibrary.wiley.com/doi/10.1002/14651858.CD001842.pub2/abstract.

#### Estrogens

This review^12^ evaluated the effects of estrogens (local or systemic) on post-menopausal women with urinary incontinence (UI), and included 34 RCTs (19,676 participants). From the comparison of estrogen versus placebo/no treatment, the following findings were observed:

- Number of participants with no improvement of urinary incontinence: use of oral systemic estrogens gave rise to a higher number of participants with no improvement (risk ratio [RR] 1.32; 95% confidence interval [CI] 1.17 to 1.48; six RCTs; 6,151 participants; low quality of evidence), while use of local estrogens gave rise to a lower number of participants with no improvement (RR 0.74; 95% CI 0.64 to 0.86; four RCTs; 213 participants; very low quality of evidence).
- Episodes of urinary incontinence over 24 hours: no difference between the groups (mean difference [MD] 0.54; 95% CI -0.5 to 1.57; two RCTs; 82 participants; low quality of evidence).
- Women with urge incontinence: no difference between use of systemic estrogens and placebo (RR 1.05; 95% CI 0.83 to 1.33; two RCTs; 89 participants; moderate quality of evidence), but lower rate with local estrogens than with placebo (RR 0.38; 95% CI 0.15 to 0.99; two RCTS; 90 participants; moderate quality of evidence).
- Women with adverse events: higher rate with use of systemic estrogens (RR 13; 95% CI 1.87 to 90.21; one RCT; 40 participants; low quality of evidence). No difference in adverse events was found with use of local estrogens, compared with placebo (RR 1.33; 95% CI 0.32 to 5.61; two studies; 144 participants; moderate quality of evidence).

The authors concluded that local estrogen treatment may improve UI, but that there was only limited evidence regarding its effects in the post-treatment period and over the long term. However, it was observed that systemic estrogen treatment might worsen incontinence. The data were insufficient to identify the optimal estrogen type and dose, and there was no direct evidence for comparing routes of administration. The risk of endometrial and breast cancer after long-term use of systemic estrogen suggested that this treatment should only be administered for limited periods, especially among women with an intact uterus. 

For further details, refer to the original abstract, available from: http://onlinelibrary.wiley.com/doi/10.1002/14651858.CD001405.pub3/abstract.

#### Serotonin-noradrenaline reuptake inhibitors (SNRIs) 

This review^13^ assessed the effects of a SNRI (duloxetine) for treating women who presented UI. Ten RCTs were included (n = 3,944), and these compared duloxetine with placebo and/or pelvic floor muscle training over periods of three to 12 weeks. The main results were:

- Number of participants with urinary incontinence at the end of the follow-up: no benefits from use of duloxetine 80 mg/day (RR 0.97; 95% CI 0.93 to 1.00; three RCTs; 1,396 participants), 40 mg/day (RR 0.89; 95% CI 0.79 to 1.01; one RCT; 255 participants) and 20 mg/day (RR 0.99; 95% CI 0.89 to 1.09; one RCT; 260 participants).
- Number of participants without improvement during treatment: lower through use of duloxetine 80 mg/day (RR 0.74; 95% CI 0.68 to 0.81; four RCTs; 1,733 participants), 40 mg/day (RR 0.64; 95% CI 0.45 to 0.90; one RCT; 67 participants) and 20 mg/day (RR 0.55; 95% CI 0.40 to 0.75; two RCTs; 160 participants).
- Adverse events: more frequent through use of duloxetine 80 mg/day (RR 1.25; 95% CI 1.14 to 1.36; six RCTs; 1,879 participants).

These results showed that duloxetine could improve symptoms but failed to show any objective improvements in cure rate. The number of participants presenting adverse events was higher in the duloxetine group. Further studies comparing duloxetine and other SNRIs with other treatments are needed in order to reach additional conclusions.

For further details, refer to the original abstract, available from: http://onlinelibrary.wiley.com/doi/10.1002/14651858.CD004742.pub2/abstract.

### Behavioral methods

#### Biofeedback

This review^14^ assessed the effects of pelvic exercises using different types of feedback and biofeedback among women with urge incontinence and mixed urinary incontinence. Twenty-four trials (n = 1,583) were included and the main results were:

- Pelvic floor muscle training (PFMT) with biofeedback versus PFMT alone: PFMT with biofeedback was statistically significantly better for improving women’s perception of change in incontinence (RR 0.75; 95% CI 0.66 to 0.86; seven RCTs; 520 participants) and women’s satisfaction (RR 0.65; 95% CI 0.46 to 0.90; three RCTs; 294 participants). There was also a marginally significant result regarding reduction of the number of leakage episodes (MD -0.12; 95% CI -0.22 to -0.01; eight RCTs; 532 participants), but this result might not be clinically relevant.
- PFMT with feedback versus PFMT alone: The group that received feedback had better perception of change in incontinence (RR 0.53; 95% CI 0.37 to 0.78; one RCT; 122 participants) and greater satisfaction (RR 0.33; 95% CI 0.16 to 0.66; one RCT; 166 participants).

Other comparisons were made, but the effects of biofeedback/feedback interventions could not be individually interpreted. The authors of this SR concluded that feedback or biofeedback might provide benefits when added to PFMT in treating urinary incontinence. However, no assessment of quality of evidence was made and no data for safety assessment were provided in the RCTs included. 

For further details, refer to the original abstract, available from: http://onlinelibrary.wiley.com/doi/10.1002/14651858.CD009252/abstract.

#### Bladder training

This review^15^ assessed bladder training, defined as a behavioral technique in which the patient is trained to resist the first urge to urinate and to refrain from urinating until a planned scheduled time. Eight RCTs (n = 858, predominantly female) were included and the main findings were:

- Bladder training versus oxybutynin:
  - Patient’s perception of cure at six months: better in bladder training group (RR 1.69; 95% CI 1.21 to 2.34; one RCT; 81 participants).
  - Quality of life (general physical measurement): better in bladder training group (weighted mean difference [WMD] 9.00; 95% CI 1.64 to 16.36; one RCT; 57 participants).
- Bladder training versus imipramine plus flavoxate:
  - Patient’s perception of cure immediately after treatment: better in bladder training group (RR 1.50; 95% CI 1.02 to 2.21; one RCT; 50 participants).
- Bladder training plus pelvic exercises plus biofeedback versus pelvic exercises plus biofeedback:
  - Patient’s perception of improvement: better with bladder training (RR 1.18; 95% CI 1.01 to 1.39; one RCT; 125 participants).

All the results of this systematic review were based on poorly reported low-quality RCTs with small sample sizes. The main outcomes were subjective and not pre-specified. Therefore, conclusions regarding the effectiveness and safety of bladder training must await further studies with good methodological and reporting qualities. 

For further details, refer to the original abstract, available from: http://onlinelibrary.wiley.com/doi/10.1002/14651858.CD001308.pub2/abstract.

#### Habit retraining

This review^16^ assessed the effects of habit retraining, which consists of assistance for toileting provided by a caregiver, with the aim of management of UI. Four RCTs (n = 378) were included, and most of the participants were elderly women who were physically and/or cognitively impaired, and dependent on caregivers. All the RCTs had important methodological limitations and the sparseness of the reported data and the clinical diversity between the studies prevented any quantitative synthesis. Until further studies are available, no judgment regarding the effectiveness and safety of habit retraining can be made. 

For further details, refer to the original abstract, available from: http://onlinelibrary.wiley.com/doi/10.1002/14651858.CD002801.pub2/full.

#### Lifestyle interventions

This review^17^ assessed the effects of low-cost, non-invasive lifestyle interventions, such as

- dietary changes
- weight loss
- smoking cessation
- physical activity and
- fluid intake in relation to management of UI. Eleven RCTs were included (n = 5,974; mostly female participants).

The following results were found regarding comparison of weight loss programs versus any other intervention:

- Patient’s subjective impression of improvement: better in the weight loss program group (RR 1.40; 95% CI 1.14 to 1.71; one RCT; 304 participants; low quality of evidence).
- Cure rates: no difference between groups (RR 1.11; 95% CI 0.91 to 1.37; one RCT; 738 participants; low quality of evidence).
- Frequency of incontinence episodes per week: no difference between groups (RR 0.88; 95% CI 0.78 to 1.00; one RCT; 2,739 participants, very low quality of evidence).

All the trials included were judged to present high risk of bias. Taking into account the quality of the body of evidence relating to weight loss programs and the lack of high quality RCTs, no solid conclusion can be drawn until new studies are available. 

For further details, refer to the original abstract, available from: http://onlinelibrary.wiley.com/doi/10.1002/14651858.CD003505.pub5/abstract.

#### Prompted voiding

This review^18^ assessed the effects of prompted voiding (a behavioral technique in which the patients receive verbal toilet reminders) for elderly people with UI. Nine RCTs were included (n = 674, the majority of whom were women). The prompted voiding group presented better results for the following outcomes:

- Reduction of the proportion of hourly checks that found wetness (mean difference (MD) 12%; 95% CI -18.79% to -5.21%; one RCT; 148 participants).
- Fewer episodes of incontinence over 24 hours (MD -0.92; 95% CI -1.32 to -0.53; two RCTs; 257 participants).

No statistical difference between the groups were found regarding the number of people with no improvement in wet episodes (OR 0.60; CI 95% 0.29 to 1.26; one RCT; 133 participants). Many of the RCTs included did not provided minimum statistical data for analysis. Also, the clinical outcomes assessed in these RCTs were too diverse and poorly reported. The small sample size and poor methodological quality of the RCTs were an obstacle to drawing any recommendations for practice. Further studies investigating prompted voiding are needed in order to evaluate the safety and effectiveness of this intervention. 

For further details, refer to the original abstract, available from: http://onlinelibrary.wiley.com/doi/10.1002/14651858.CD002113/abstract.

#### Timed voiding

This review^19^ evaluated the effects of timed voiding for management of UI among elderly women. Two RCTs of poor methodological quality (n = 298) were included and these assessed the effects of timed voiding combined with the usual care (estrogens, propantheline and flavoxate). Therefore, the extent of the effects of timed voiding alone could not be assessed. 

For further details, refer to the original abstract, available from: http://onlinelibrary.wiley.com/doi/10.1002/14651858.CD002802.pub2/abstract.

### Miscellaneous interventions

#### Absorbent products

This review^20^ evaluated the effectiveness of different types of absorbent products for moderate-to-heavy incontinence. Two RCTs were included (n = 185). Fourteen comparisons of absorbent products were made, including disposable insert pads, disposable diapers, T-shaped diapers, disposable pull-ups and washable diapers. The results were sparse and based on only two low-quality RCTs. For most of the comparisons, no difference in the effects was found. Furthermore, for those in which a difference was found, the low quality of the studies precluded any practical recommendation. 

For further details, refer to the original abstract, available from: http://onlinelibrary.wiley.com/doi/10.1002/14651858.CD007408/abstract.

#### Acupuncture

This review^21^ evaluated the benefits and harms of acupuncture among adults with UI. It included one RCT (n = 60 women) that compared acupuncture versus midodrine:

- Improvement of symptoms (subjective assessment): better with acupuncture (RR 2.20; 95% CI 1.27 to 3.81; one RCT; 60 participants).
- Cure rate: no difference between groups (RR 2.00; 95% CI 0.40 to 10.11; one RCT; 60 participants).

Because this review included only one small head-to-head study with low methodological quality and poor reporting, no conclusions can be reached regarding the efficacy and safety of acupuncture among UI patients. 

For further details, refer to the original abstract, available from: http://onlinelibrary.wiley.com/doi/10.1002/14651858.CD009408.pub2/abstract.

#### Mechanical devices

The objective of this review^22^ was to assess the use of mechanical devices for management of adult female UI. Eight RCTs were included (n = 787), but no solid conclusions could be drawn because of the lack of reporting and lack of relevant clinical outcome assessment. The RCTs included were poorly reported and no meta-analysis could be performed because of the scarcity and heterogeneous nature of the data. Until further good methodological quality RCTs are published, the uncertainty regarding the effects of mechanical devices in patients with UI will remain unresolved.

For further details, refer to the original abstract, available from: http://onlinelibrary.wiley.com/doi/10.1002/14651858.CD001756.pub6/abstract.

#### Transurethral radiofrequency collagen denaturation (TRCD)

This review^23^ evaluated the effects of TRCD in relation to incontinence among women. TRCD is a minimally invasive device-based intervention in which a low-temperature heat is applied via the urethra. One RCT (n = 142) comparing TRCD with a sham group found the following:

- Quality of life: no difference between groups (RR 1.11; 95% CI 0.77 to 1.62; one RCT; 142 participants; low quality of evidence)
- Adverse events: no difference between groups (RR 1.36; 95% CI 0.63 to 2.93; one RCT; 173 participants, low quality of evidence)
- Urinary tract infection: no difference between groups (RR 0.95; CI 95% 0.24 to 3.86; one RCT; 173 participants; low quality of evidence).

The authors concluded that there was insufficient evidence to be able to state that TRCD improves quality of life among patients with UI. 

For further details, refer to the original abstract, available from: http://onlinelibrary.wiley.com/doi/10.1002/14651858.CD010217.pub2/abstract.

#### Treatment of UI after stroke 

This review^24^ evaluated different treatments for UI after stroke and included 12 RCTs (n = 724 participants). The interventions considered were the following: behavioral interventions (three RCTs), specialized professional input interventions (two RCTs), complementary therapy interventions (three RCTs) and pharmacotherapy and hormonal interventions (three RCTs). Most of the RCTs included had small sample sizes and were poorly reported. The main findings were:

- Meclofenoxate versus placebo: reduction in the perception of urinary symptoms through use of meclofenoxate (RR 0.33; 95% CI 0.18 to 0.62; one RCT; 80 participants).
- Acupuncture versus usual care/no intervention: reduction in the number of participants with incontinence after treatment in the acupuncture group (RR 0.44; 95% CI 0.23 to 0.86; three RCTs; 219 participants).

The authors concluded that there was insufficient evidence to guide UI management after stroke. 

For further details, refer to the original abstract, available from: http://onlinelibrary.wiley.com/doi/10.1002/14651858.CD004462.pub3/abstract.

#### Urethral injection therapy

This review^25^ evaluated the efficacy and safety of injections of periurethral or transurethral bulking agents that had the aim of creating artificial cushioning in the area around the urethra. It included 14 RCTs (n = 2,004 women) and the main results were:

- Injection versus conservative treatment: the injection treatment group had a higher number of participants with improvement of continence than did the conservative treatment group (RR 0.70; 95% CI 0.52 to 0.94; one RCT; 45 participants) and it achieved a statistically significant improvement of quality of life (MD 0.54; 95% CI 0.16 to 0.92; one RCT; 45 participants).
- Injection versus surgical management: the surgical group had higher rates of symptom improvement in two trials: RR 4.77; 95% CI 1.96 to 11.64; one RCT; 45 participants in one study; and RR 1.69; 95% CI 1.02 to 2.79; one RCT; 54 participants in the other study.

The results from the remaining RCTs included were poorly reported and no data could be pooled because of the lack of relevant outcome results and because of the heterogeneity between studies. Until further high-quality studies are available, the benefits of urethral injection therapy in patients with urinary incontinence will remain a matter that needs to be resolved.

For further details, refer to the original abstract, available from: http://onlinelibrary.wiley.com/doi/10.1002/14651858.CD003881.pub4/abstract.

#### Weighted vaginal cones

This review^26^ evaluated the efficacy and safety of use of weighted vaginal cones for women with urinary incontinence and it included 23 RCTs (n = 1,806). The following results were found:

- Weighted vaginal cones versus no treatment: the intervention group had a lower number of patients with urinary incontinence at the end of the follow-up (RR 0.84; 95% CI 0.76 to 0.94; four RCTs; 375 participants).
- Weighted vaginal cones versus pelvic exercises: no difference between the groups regarding subjective assessment of cure (RR 1.01; 95% CI 0.91 to 1.13; five RCTs; 338 participants).
- Weighted vaginal cones versus electrostimulation: no difference between the groups regarding subjective assessment of cure (RR 1.26; 95% CI 0.85 to 1.87; three RCTs; 151 participants).

The authors concluded that these findings suggested that cones were a better option than no treatment. However, no assessment of the quality of the body of evidence was made. More high-quality trials are necessary in order to fully evaluate the possible benefits and adverse events of use of weighted cones for treatment of urinary incontinence, preferably with larger sample sizes and using standard relevant outcomes.

For further details, refer to the original abstract, available from: http://onlinelibrary.wiley.com/doi/10.1002/14651858.CD002114.pub2/abstract.

### Pelvic floor muscle training (PFMT) interventions 

Four reviews^27^^,^^28^^,^^29^^,^^30^ assessed the efficacy and safety of PFMT for management of UI (stress, urge and mixed) in women.

One review^27^ compared PFMT combined with active treatment versus the same active treatment alone, and included 13 trials (n = 1,164). The key outcomes considered by the review authors were not assessed in the majority of the RCTs included. The main results reported were:

- PFMT plus electrical stimulation versus electrical stimulation alone: no difference between groups regarding cure or improvement of symptoms (RR 2.06; 95% CI 0.79 to 5.38; two RCTs; 56 participants; very low quality of evidence).
- PFMT plus heat and steam-generating sheet *versus* heat and steam-generating sheet alone: the group that received PFMT had higher likelihood of participants presenting cure or symptom improvement (RR 2.38; 95% CI 1.19 to 4.73; one RCT; 74 participants; moderate quality of evidence).
- PFMT plus vaginal cones versus vaginal cones alone: no difference between groups (RR 1.27; 95% CI 0.94 to 1.71; one RCT; 34 participants; very low quality of evidence).

The adverse events were only evaluated in one RCT in which PFMT in combination with drug therapy was assessed, and this did not find any statistical difference (RR 0.84; 95% CI 0.45 to 1.60; one RCT; 162 participants; very low quality of evidence). In the light of these findings, the authors concluded that there was insufficient evidence to state what the effects of addition of PFMT to treatments for urinary incontinence were.

For further details, refer to the original abstract, available from: http://onlinelibrary.wiley.com/doi/10.1002/14651858.CD010551.pub3/abstract.

The second review^28^ included 21 RCTs (n = 1,281 participants) that compared PFMT with no treatment, placebo or sham. The main results were:

- Cure at the end of the follow-up: higher likelihood in the PFMT group (RR 8.38; 95% CI 3.68 to 19.07; four RCTs; 165 participants; high quality of evidence).
- Symptom improvement: higher likelihood in the PFMT group (RR 17.33; 95% CI 4.31 to 69.64; two RCTs; 121 participants; moderate quality of evidence).

The authors concluded that these findings supported a recommendation to use PFMT as first-line conservative therapy for women with urinary incontinence. 

For further details, refer to the original abstract, available from: http://onlinelibrary.wiley.com/doi/10.1002/14651858.CD005654.pub3/abstract.

Other comparisons were made, but the data were sparse and based on low-quality RCTs. Moreover, there was no assessment of the quality of the body of evidence for any comparison. The authors concluded that currently there was insufficient evidence to make any strong recommendations about the best approach to use for PFMT.

For further details, refer to the original abstract, available from: http://onlinelibrary.wiley.com/doi/10.1002/14651858.CD009508/abstract.

The fourth review^30^ assessed the effects of PFMT for prevention and treatment of UI among antenatal and postnatal women. Twenty-two RCTs (n = 8,485) were included and the main findings were:

- Incidence of incontinence over the first six months after delivery (prevention): lower likelihood among pregnant women with intensive antenatal PFMT than among those with postnatal PFMT (RR 0.71; 95% CI 0.54 to 0.95; five RCTs; 673 participants).
- Rate of incontinence 12 months after delivery (treatment): no difference in likelihood between pregnant women with intensive postnatal PFMT and those who received intensive antenatal PFMT (RR 0.60; 95% CI 0.35 to 1.03; three RCTs).

The authors concluded that there was some evidence to support the use of PFMT among women who were having their first baby, to prevent UI over the first six months after delivery. However, there was no appraisal of the quality of the evidence, and the clinical and methodological heterogeneity among the studies included prevented other pooled analyses.

For further details, refer to the original abstract, available from: http://onlinelibrary.wiley.com/doi/10.1002/14651858.CD007471.pub2/abstract.

## DISCUSSION

This review included 20 Cochrane systematic reviews that assessed non-surgical interventions for women with urinary incontinence. The studies included evaluated pharmacological interventions (three SRs), behavioral interventions (six SRs), miscellaneous interventions (seven SRs) and pelvic floor muscle training interventions (four SRs).

Despite the considerable numbers of studies included in many of these SRs, most of them were poorly reported with low methodological quality, which compromised confidence in the data synthesis. However, the quality of the body of evidence was only assessed in five SRs.^12^^,^^17^^,^^23^^,^^27^^,^^28^ Since the publication of the GRADE initiative,^31^ evaluation of the quality of the evidence synthetized has become recommended and, for further updates, this needs to be a priority.^31^

Regarding the clinical implications, the only systematic reviews that presented moderate to high quality of evidence were those relating to use of pelvic floor muscle training among women with urinary incontinence. On the other hand, the quality of evidence reported was very low to moderate in relation to use of estrogen for treating and preventing urinary incontinence. Moreover, the outcomes reported from all other interventions presented very low to low quality of evidence. The results presented in Table 2 may provide some guidance for using some of the interventions evaluated in this overview of systematic reviews, but most of the findings may change through future studies.

Regarding the implications for further research, our results suggest that it is important to plan and conduct randomized controlled trials assessing non-pharmacological interventions for women with urinary incontinence strictly in accordance with rigorous protocols. The trial protocols need to be published before the study begins and the results need to be presented in accordance with the CONSORT guidelines.^32^

## CONCLUSION

This review included 20 Cochrane systematic reviews that provided evidence ranging in quality from unknown to high, in relation to non-pharmacological interventions for patients with urinary incontinence. Moderate to high quality of evidence favoring use of pelvic floor muscle training among women with urinary incontinence was found. To establish solid conclusions for all the other comparisons, further studies of good methodological quality are needed.
